# Metagenomes enriched with *Virgibacillus* are associated with a pink paste defect in an unpasteurized blue cheese

**DOI:** 10.1128/mra.00027-26

**Published:** 2026-04-30

**Authors:** Benjamin E. Wolfe

**Affiliations:** 1 Department of Biology, Tufts University 1810, Medford, Massachusetts, USA; Montana State University, Bozeman, Montana, USA

**Keywords:** cheese, metagenome, *Virgibacillus*

## Abstract

Shotgun metagenomes were used to identify microbes associated with a pink discoloration of an unpasteurized blue cheese made in the United States. Taxonomic assessments of individual reads and metagenome-assembled genomes revealed that the genus *Virgibacillus* was present in the pink paste, but not in unaffected paste.

## ANNOUNCEMENT

Microbes play essential roles in driving the quality and safety of cheeses by generating the flavors and esthetics of these products (1–3). Some smaller-scale artisan or farmstead cheeses can experience variation in product quality from one batch to another due to variation in microbes in raw ingredients, aging conditions, or other factors (4). During the production and aging of these cheeses, color defects can form in the paste or on the surface, affecting marketability of these products (5–8). Pink or red discoloration is a common pigment defect in cheeses, and it is thought that several different types of microbes, including bacteria and yeasts, can be a cause (9–11).

I used shotgun metagenomics to analyze the paste of an unpasteurized blue cheese produced in the United States that had pink discoloration across many wheels in several batches. Discoloration was only observed in the paste of the cheese and not on the cheese surface. Using a pair of sterile metal forceps, four samples (approximately 2–10 g) were taken from areas without discoloration, and four samples were taken from paste with obvious pink pigments. DNA was extracted using a Qiagen DNeasy PowerFood Microbial Kit following the manufacturer’s instructions; 30 µL of DNA (quantified with a Qubit dsDNA HS Assay Kit and ranging from 47 to 52 ng/µL) was suspended in 10 mM Tris-HCl (pH 7.5–8.0); and 1 mM EDTA buffer was used to prepare sequencing libraries using an Illumina DNA Prep Kit (with on-bead tagmentation to fragment the DNA and no size selection) and Integrated DNA Technologies 10 bp unique dual indexes for multiplexing. Libraries were sequenced on an Illumina NextSeq 2000, producing 2 × 151 bp reads with a range of 11.5 to 19.2 million reads per library. Demultiplexing, quality control, and adapter trimming were performed by bcl2fastq (2.20.0.445) with default parameters. Taxonomic assignment of reads was conducted with Kaiju v1.9.0 (12) with the National Center for Biotechnology Information (NCBI) BLAST nr + euk reference database, a low abundance filter of 0.5, and a subsample percent of 10. Metagenome-assembled genomes (MAGs) of one read library from a pink sample were assembled and analyzed as previously described (13). MAGs were assembled with MEGAHIT v1.2.9 (14), assembled contigs were binned with MaxBin2 v2.2.4, and the taxonomy of the two resulting bins was determined by the Insert Genome into SpeciesTree tool in KBase.

**Fig 1:**
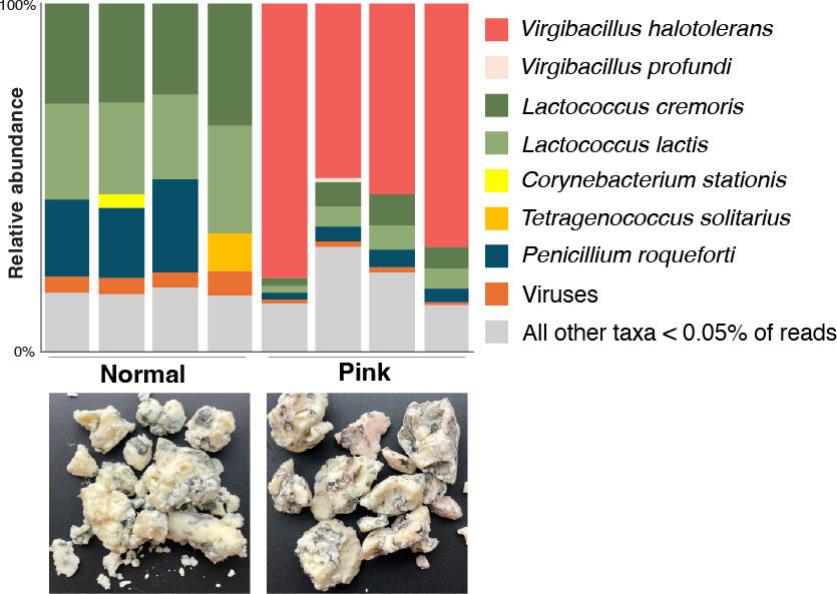
Taxonomic assignment of reads from the paste of a blue cheese that appeared normal (four bars on the left) and from paste that had pink discoloration (four bars on the right). Reads were assigned using Kaiju v1.9.0. Only reads that had a taxonomic assignment are shown.

Taxonomic assignment of reads via Kaiju identified typical blue cheese microbes (*Lactococcus* spp. and *Penicillium roqueforti*) as the dominant taxa (Fig. 1). One striking difference was a high abundance (22–54% of all reads) of the taxon *Virgibacillus halotolerans* only in the pink samples. *Virgibacillus* reads were detected in three of the four normal samples, but at very low abundance (less than <0.001% relative abundance). From the one pink sample with the highest *Virgibacillus* abundance predicted from Kaiju (46% of all reads, including unclassified reads), we constructed two MAGs, with one MAG (composed of 176 contigs) being assigned to the *Lactobacillales* (97.36% completeness, 2.88% contamination based on CheckM) and the other MAG (composed of 206 contigs) placed within *Virgibacillus* (98.25% completeness, 18.29% contamination).

While *Virgibacillus* species are not normally found in the paste of most cheeses, *V. halotolerans* was originally isolated from a spoiled (although not explicitly identified as pigmented) cheese product in Germany, and some *Virgibacillus* species are known to make pink pigments (15, 16) that may explain the defects we observed. Future experimental work is needed to determine whether and how *Virgibacillus* can cause pigment defects in cheeses.

## Data Availability

Raw metagenomic reads are available in the NCBI Sequence Read Archive with BioProject number PRJNA1396678. Individual SRA submissions are as follows: B1 (pink, SRX31662792), B2 (pink, SRX31662793), B3 (normal, SRX31662794), B4 (normal, SRX31662795), B5 (pink, SRX31662796), B6 (pink, SRX31662797), B7 (normal, SRX31662798), and B8 (normal, SRX31662799). The two MAGs have been deposited in NCBI as B5_MAG_Bin1 (Virgibacillus sp. SAMN57030317) and B5_MAG_Bin2 (Lactobacillales SAMN57030318).
